# Spontaneous Resolution of Mandibular Incisor Gingival Recession Following Orthodontic Torque Correction With Fixed Appliances: A Case Report

**DOI:** 10.7759/cureus.111208

**Published:** 2026-06-20

**Authors:** Mohamed Drissi Lahsini, Nabil Loudini, Hicham Benyahia, Fatima Zaoui

**Affiliations:** 1 Orthodontics and Dentofacial Orthopaedics, Faculty of Dental Medicine, Mohammed V University, Rabat, MAR

**Keywords:** fixed appliance, gingival recession, interdisciplinary care, orthodontic torque, spontaneous resolution

## Abstract

Gingival recession associated with excessive vestibular torque of mandibular incisors represents a significant periodontal challenge. This case report presents a non-surgical management strategy for an isolated deep recession in an adolescent patient. A 12-year-old female patient presented with severe mandibular anterior crowding and a 3 mm isolated labial gingival recession on tooth 31 (Cairo RT1/Miller Class I), secondary to severe vestibular root displacement. Treatment was carried out using a full fixed appliance (0.022-inch slot, Roth prescription) over 24 months. Individualized lingual root torque couples were progressively incorporated into 0.019 × 0.025-inch stainless steel (SS) finishing wires to relocate the root within the alveolar housing, strictly avoiding any mucogingival surgery. Significant spontaneous coronal migration of the gingival margin was achieved, reducing the recession from 3 mm to 1 mm, without root resorption. In adolescent patients presenting with RT1 recessions secondary to crown-vestibular malposition, early non-surgical orthodontic torque correction can result in significant tissue recovery and should be considered a reliable first-line therapeutic approach.

## Introduction

Gingival recession, defined as the apical displacement of the gingival margin below the cemento-enamel junction (CEJ), is a multifactorial condition affecting patients of all ages, including adolescents undergoing orthodontic treatment [1]. Its clinical significance encompasses esthetic concerns, dentinal hypersensitivity, and the risk of progressive attachment loss.

Among local etiological factors, excessive vestibular inclination of mandibular incisors, referred to as abnormal root torque, is a recognized predisposing mechanism for labial gingival recession. When the radicular third is displaced beyond the buccal alveolar plate, the overlying tissue, particularly in patients with a thin biotype, becomes vulnerable to recession [2, 3]. The mandibular anterior region is especially susceptible due to the naturally thin cortical bone of the symphysis [4, 5].

Management has traditionally relied on a staged interdisciplinary protocol: torque correction to improve the anatomical environment, followed by mucogingival surgery [6, 7]. However, in cases classified as Cairo RT1 or Miller Class I with a favorable gingival phenotype, orthodontic torque correction alone may result in spontaneous recession resolution [8, 9].

This case report describes the significant spontaneous resolution of a 3 mm labial gingival recession on tooth 31 following correction of vestibular torque with a full fixed orthodontic appliance in a 12-year-old patient presenting with anterior crowding.

## Case presentation

Patient history and chief complaint

A 12-year-old female patient with no relevant medical or dental history was referred to the Department of Orthodontics, Faculty of Dental Medicine, Mohammed V University, Rabat, Morocco. Her chief complaint was anterior mandibular crowding. No previous orthodontic treatment had been undertaken. Written informed consent was obtained from the patient's legal guardians.

Clinical examination 

Extraoral examination revealed a convex facial profile with a Class II skeletal pattern and a normodivergent facial type (Figure 1).

**Figure 1:**
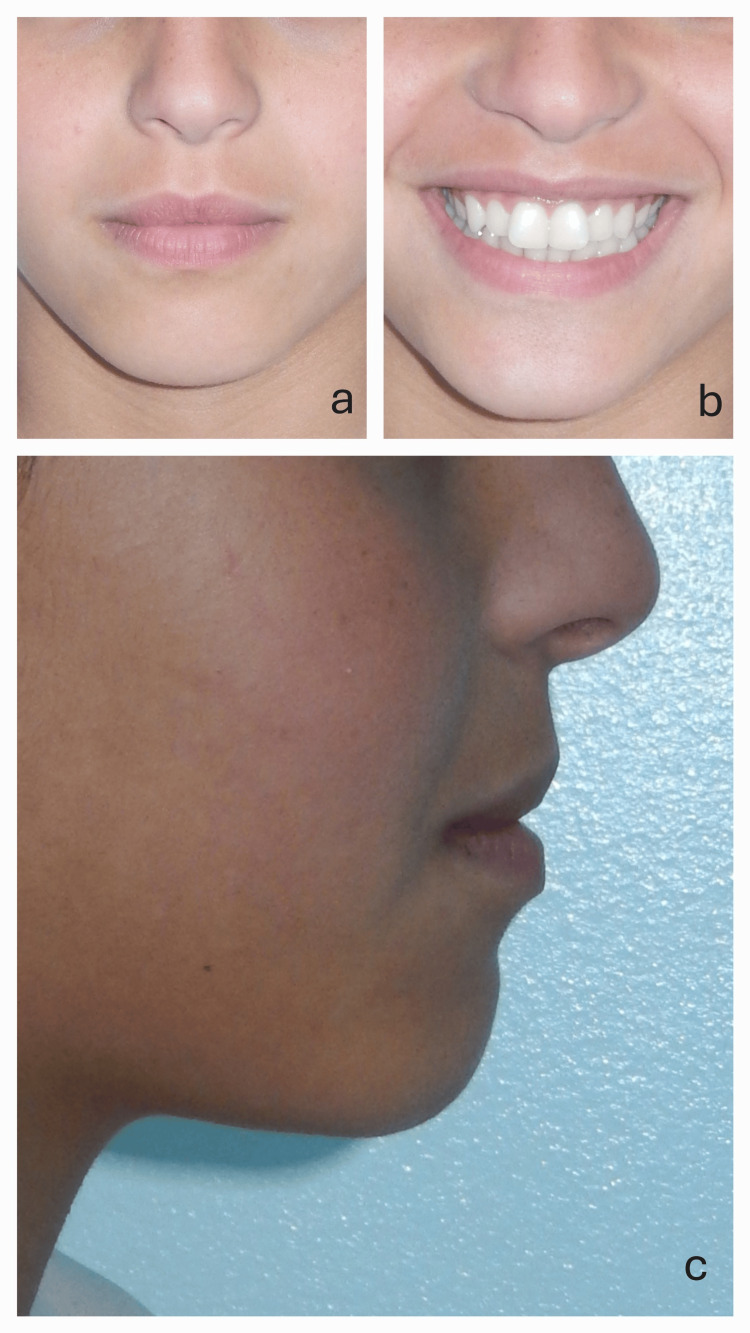
Pretreatment extraoral facial photographs: (a) frontal view at rest, (b) frontal smiling view, and (c) lateral profile view.

Intraoral examination confirmed Angle Class I dental occlusion with significant mandibular anterior crowding. Tooth 31 was labially displaced with marked vestibular root torque. Periodontal examination revealed a 3 mm labial recession at tooth 31, classified as Cairo RT1/Miller Class I, with a thin gingival phenotype (Figure 2).

**Figure 2:**
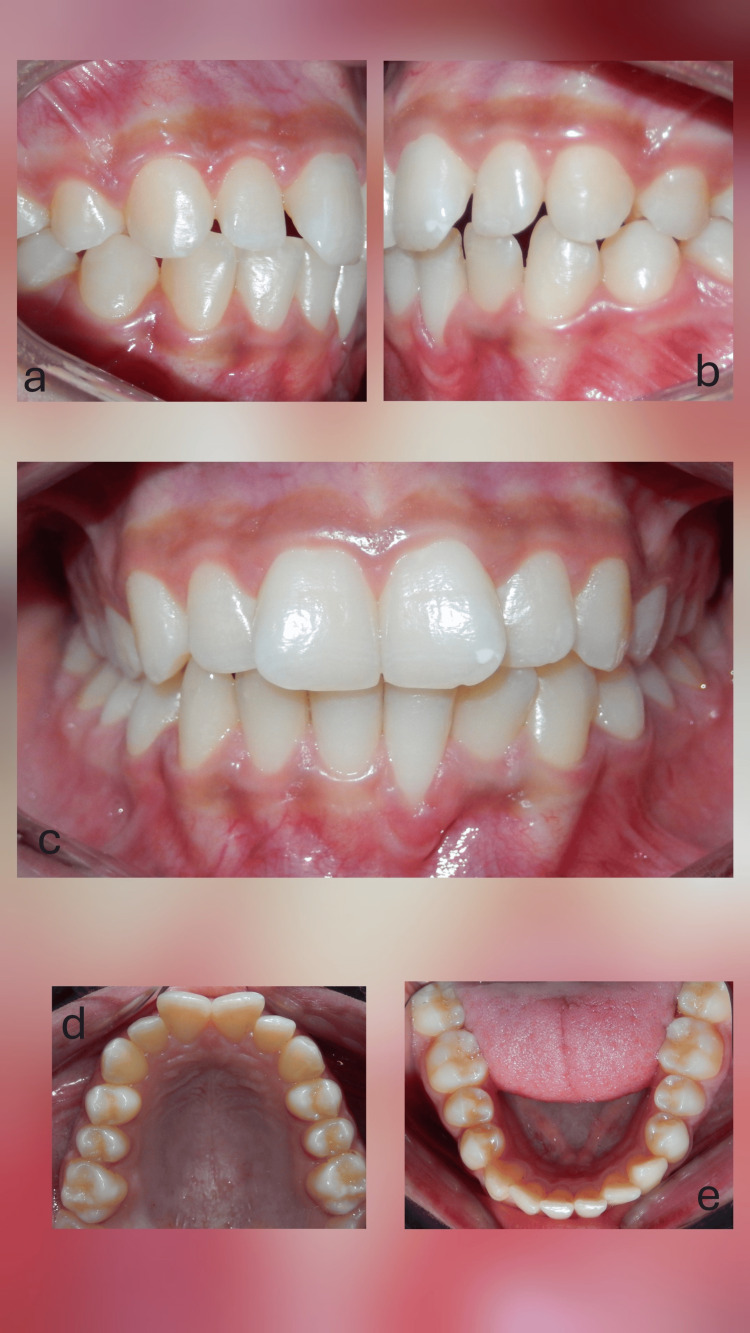
Pretreatment intraoral photographs: (a) right lateral view, (b) left lateral view, (c) frontal view showing severe labial displacement and 3 mm gingival recession on tooth 31, (d) maxillary occlusal view, and (e) mandibular occlusal view showing anterior crowding. Note the severe labial displacement and 3 mm gingival recession on tooth 31, associated with a thin gingival phenotype.

Radiographic examination 

The panoramic radiograph confirmed normal alveolar bone levels and the absence of periapical pathology. The lateral cephalogram showed a convex facial profile with a retrusive maxilla, a well-positioned mandible, proclined upper and lower incisors, and a normodivergent facial pattern with no vertical excess (Figure 3).

**Figure 3:**
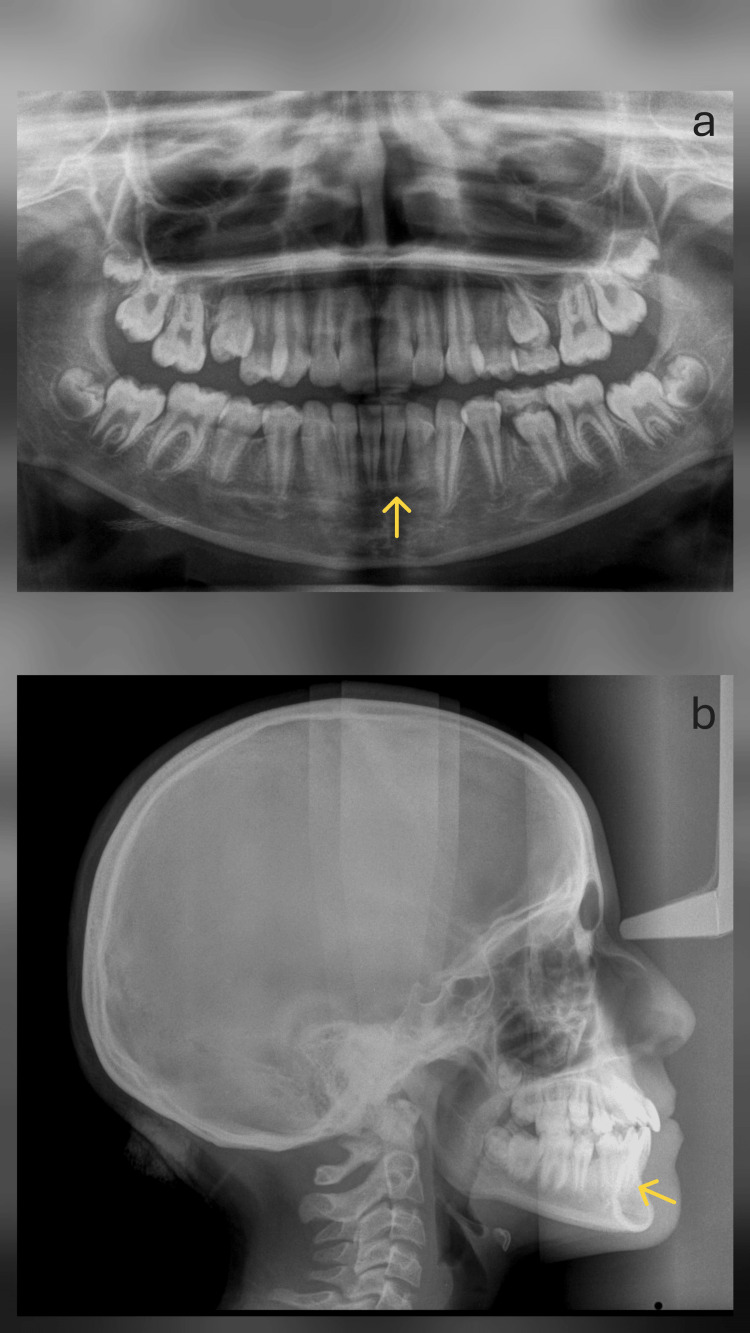
Pre-treatment radiographic records: (a) panoramic radiograph showing normal alveolar bone levels (arrow points to the tooth 31 region), and (b) lateral cephalogram demonstrating excessive incisor proclination (arrow highlights the lower incisor position within the symphysis cortical bone).

Lateral cephalometric analysis revealed a mild skeletal Class II tendency with a retrusive maxilla (Sella-Nasion-A point angle (SNA) = 77.1°, Sella-Nasion-B point angle (SNB) = 76.6°, A point-Nasion-B point angle (ANB) = 0.5°, Wits = -1.6 mm). The facial pattern was normodivergent (Frankfort mandibular plane angle (FMA) = 25.8°). Dental analysis revealed proclined and protruded mandibular incisors (incisor mandibular plane angle (IMPA) = 96.1°; lower incisor to Nasion-B point line (L1-NB) = 26.3°/6.9 mm), excessive upper incisor proclination (Upper incisor to Nasion-A point line (U1-NA) = 30.4°/10.7 mm), and a reduced interincisal angle (upper incisor-lower incisor angle (U1-L1) = 122.9°), all consistent with bimaxillary dentoalveolar protrusion superimposed on the underlying skeletal discrepancy (Table 1).

**Table 1:** Pretreatment and post-treatment cephalometric values. SNA: Sella-Nasion-A point angle; SNB: Sella-Nasion-B point angle; ANB: A point-Nasion-B point angle; U1-NA: Upper incisor to Nasion-A point line; L1-NB: Lower incisor to Nasion-B point line; U1-L1: Upper incisor-lower incisor angle; FMA: Frankfort mandibular plane angle; IMPA: Incisor mandibular plane angle.

Parameter	Norm	Pretreatment	Post-treatment
SNA (°)	82° ± 2°	77.1°	80.6°
SNB (°)	80° ± 2°	76.6°	76.7°
ANB (°)	2° ± 2°	0.5°	3.9°
Wits appraisal (mm)	0 mm ± 2 mm	-1.6 mm	-0.2 mm
U1-NA (°)	22°	30.4°	22.6°
U1-NA (mm)	4 mm	10.7 mm	2.8 mm
L1-NB (°)	25°	26.3°	24.7°
L1-NB (mm)	4 mm	6.9 mm	4.2 mm
U1-L1 (°)	131°	122.9°	128.7°
FMA (°)	25° ± 3°	25.8°	25.5°
IMPA (°)	90° ± 5°	96.1°	93.5°

The treatment objectives were to: (1) correct mandibular anterior crowding; (2) correct the vestibular root torque of tooth 31; (3) establish Class I molar and canine relationships with normal overjet and overbite; (4) achieve spontaneous resolution of the recession on tooth 31; and (5) monitor periodontal status throughout active treatment.

Mini Diamond brackets (Ormco®, Glendora, CA, USA; 0.022-inch slot, Roth prescription) were bonded on both arches. The treatment was conducted without extraction of premolars or wisdom teeth.

Treatment followed four sequential stages. The first stage, alignment and leveling, was carried out using a progressive archwire sequence: 0.014-inch Nickel-titanium (NiTi) round → 0.016 × 0.022-inch NiTi → 0.016 × 0.022-inch stainless steel (SS). This phase allowed progressive alignment of the mandibular incisors and resolution of crowding.

The second stage focused on torque correction. Individualized lingual root torque was applied to tooth 31 through the incorporation of a torque couple in the 0.019 × 0.025-inch SS finishing wires, without the need for auxiliary torquing devices. Torque expression was carefully monitored at each visit to avoid over-penetration of the radicular surface. Recession depth was recorded systematically at every appointment.

The third stage, arch coordination, aimed to establish Class I molar and canine relationships with normal overjet and overbite.

The fourth stage, finishing and retention, allowed refinement of occlusal contacts. At debonding, a passively adapted mandibular bonded lingual retainer, extending from canine to canine, was placed to avoid any vestibular mechanical stress on the periodontium of tooth 31. Active treatment lasted 24 months, with regular periodontal monitoring throughout each phase.

At the end of active treatment, significant resolution of the labial gingival recession on tooth 31 was achieved, reducing the recession from 3 mm to 1 mm and leaving a minimal residual recession of 1 mm apical to the CEJ. Probing depths were within normal limits (≤2 mm). Mandibular crowding was fully resolved with maintained Class I relationships. Near-end orthodontic radiographs confirmed satisfactory root parallelism and the absence of root resorption before appliance removal, as illustrated in the post-treatment extraoral views (Figure 4), intraoral views (Figure 5), and near-end orthodontic radiographs (Figure 6).

**Figure 4:**
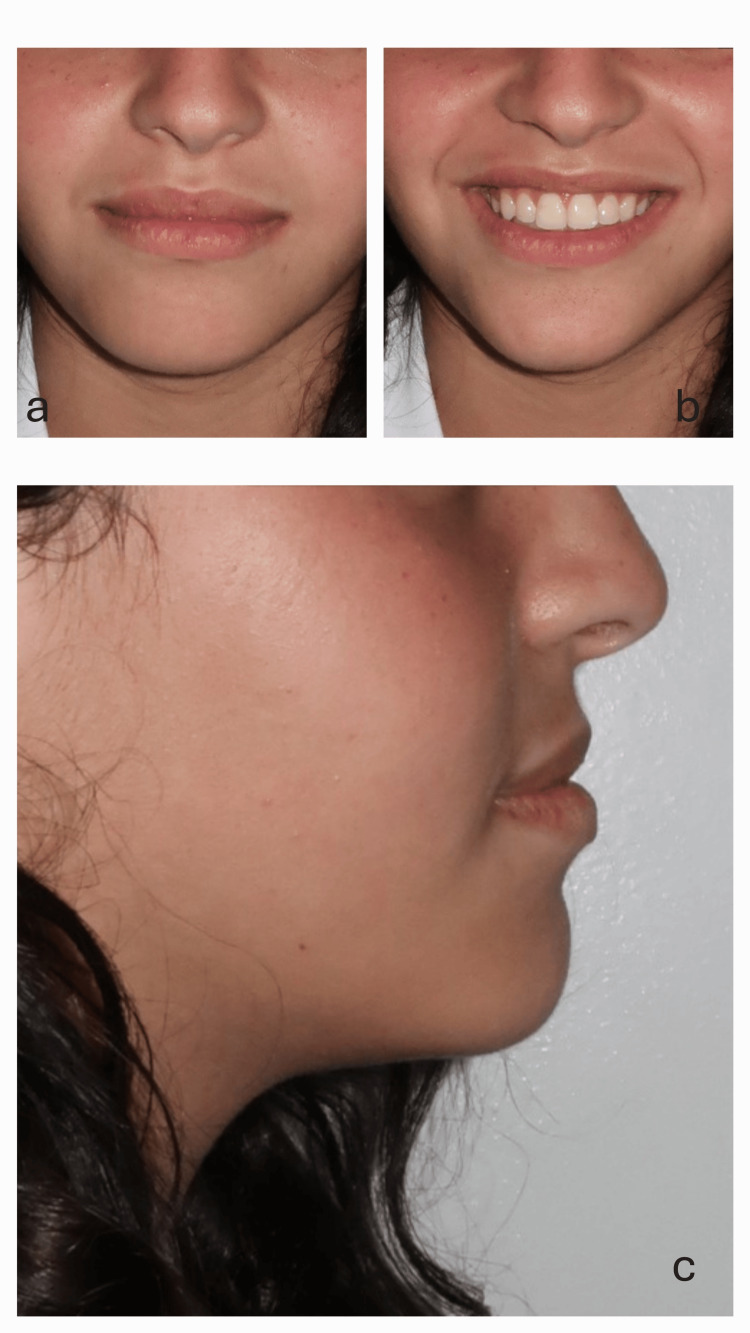
Post-treatment extraoral photographs: (a) frontal view at rest, (b) frontal smiling view showing improved smile aesthetics, and (c) lateral profile view with maintained facial balance.

**Figure 5:**
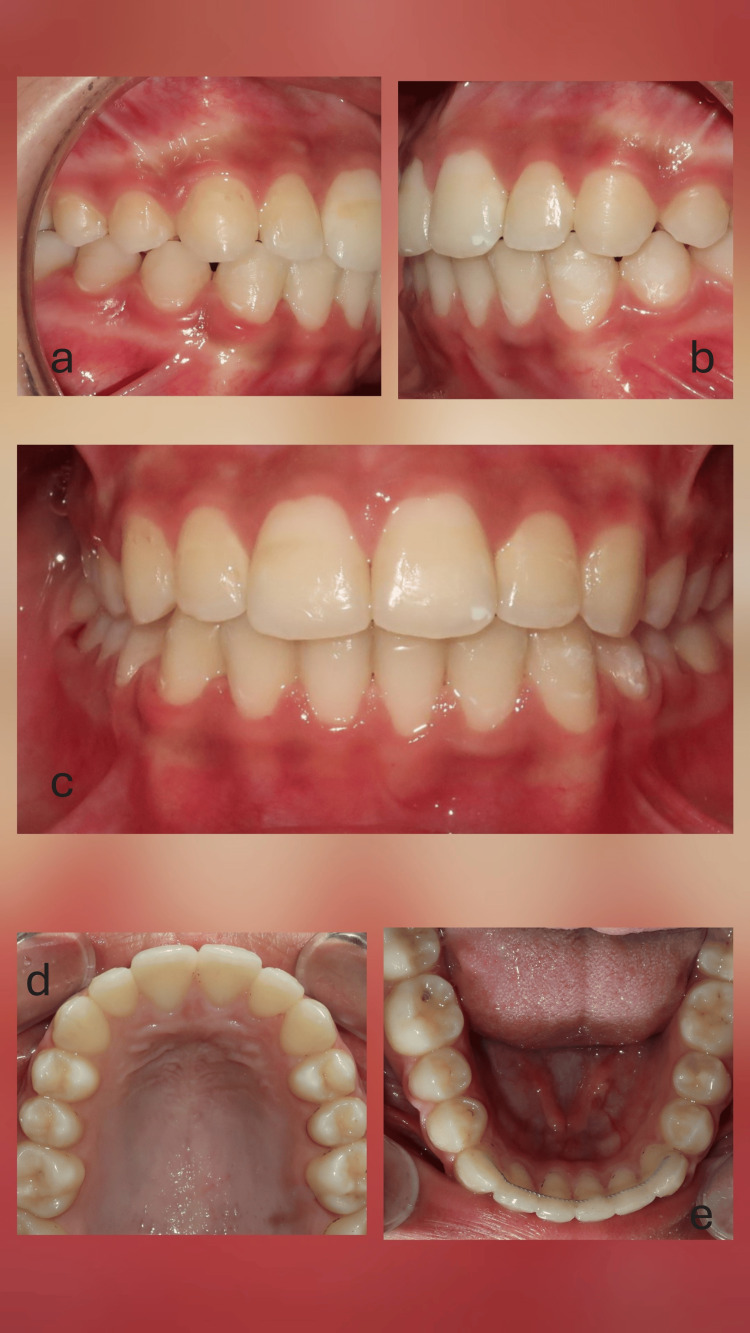
Post-treatment intraoral photographs: (a) right lateral view, (b) left lateral view showing Class I canine and molar relationships, (c) frontal view showing resolved crowding and a healthy gingival margin on tooth 31 with 1 mm residual recession, (d) maxillary occlusal view, and (e) mandibular occlusal view with the bonded lingual retainer in situ.

**Figure 6:**
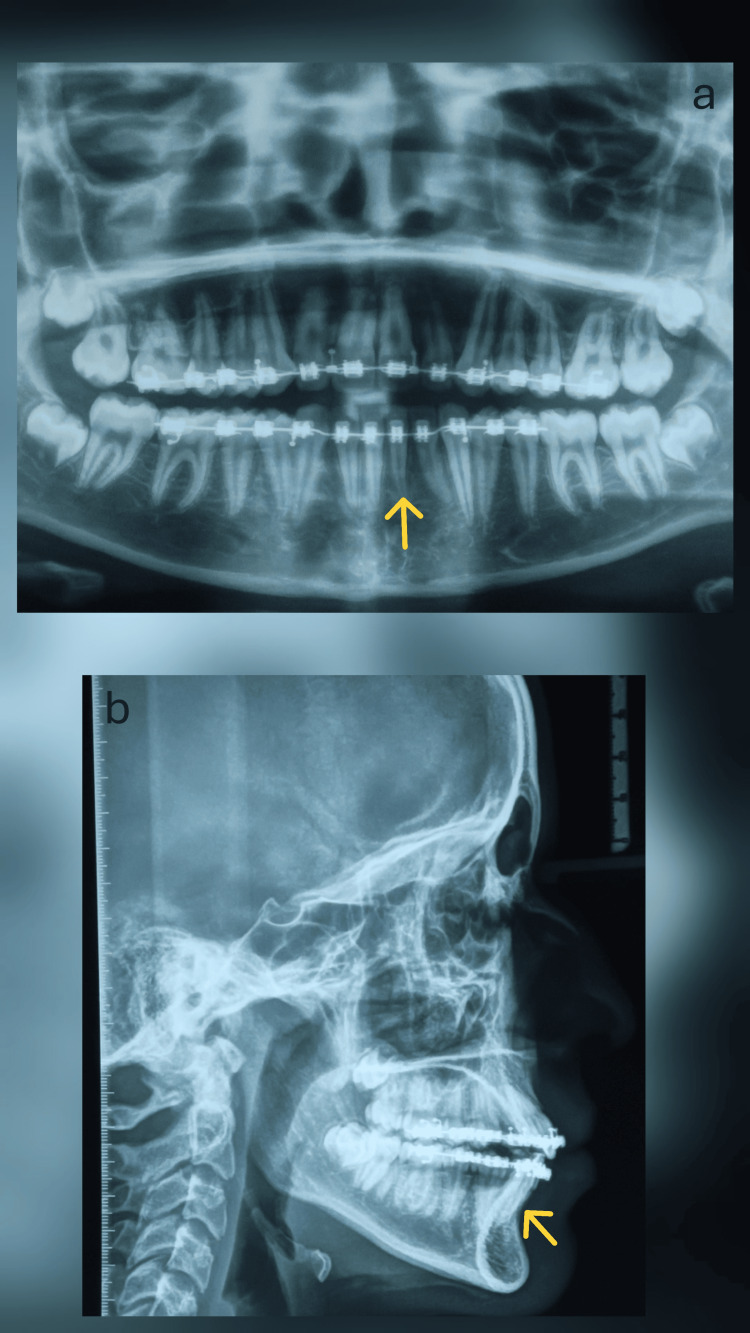
Near-end treatment radiographic records (pre-debonding evaluation): (a) panoramic radiograph confirming satisfactory root parallelism and the absence of root resorption, with the arrow pointing to tooth 31, and (b) lateral cephalogram showing the corrected mandibular incisor inclination and root torque, with the arrow highlighting the repositioned root within the alveolar housing.

## Discussion

This case demonstrates the significant spontaneous resolution of labial gingival recession on tooth 31 following vestibular torque correction with a conventional fixed appliance, without any mucogingival surgical procedure. This outcome is clinically meaningful, as it avoids surgical morbidity and associated complications in a young patient.

The pathophysiological mechanism is well established. Excessive vestibular root torque displaces the radicular surface toward or through the labial cortical plate, creating a dehiscence that deprives the overlying soft tissue of bony support [2,3,10]. Correcting this malposition by delivering precise lingual root torque repositions the root back within the alveolar housing, restoring periosteal attachment and allowing the gingival margin to migrate coronally [4,5,8]. In adolescent patients, the high bone remodeling potential may contribute to the significant extent of the tissue response observed [11].

The Cairo RT1/Miller Class I classification was the critical selection criterion predicting a favorable non-surgical outcome [12]. Similar observations using customized lingual appliances (CCLA) achieved an average recession reduction of 4.7 mm without surgery [9], and clear aligner protocols with torque overcorrection have been reported with comparable results [8]. Fadda M et al. [6] demonstrated that even in deeper recessions (>4 mm, RT3), pre-surgical torque correction systematically reclassifies the recession to RT1/RT2, improving surgical outcomes and further supporting the central role of torque management.

The use of a conventional fixed appliance with 0.022-inch Roth brackets offers reliable 3D root position control through full-size stainless steel archwires, without the torque expression limitations of clear aligner therapy [13-15]. This case also illustrates the clinical relevance of fixed appliances for complex biomechanical objectives in the anterior segment.

Limitations include the single-case design and the absence of long-term follow-up data. Recurrence remains a risk if the retainer indirectly generates vestibular stress on tooth 31. Future prospective studies with cone-beam computed tomography (CBCT) volumetric analysis and larger cohorts are needed.

## Conclusions

The present case demonstrates that orthodontic correction of excessive vestibular root torque with a full fixed appliance can achieve significant spontaneous resolution of associated labial gingival recession (Cairo RT1) on a mandibular central incisor in a 12-year-old patient, without mucogingival surgery. Success was conditioned by early interdisciplinary assessment, careful biomechanical sequencing, and appropriate patient selection based on recession classification and gingival phenotype. Furthermore, strict and regular periodontal monitoring throughout active orthodontic treatment is paramount to ensure that biomechanical forces do not jeopardize the fragile marginal periodontium. Torque correction should be considered a first-line non-surgical approach in adolescent patients with recession associated with vestibular incisor displacement.
